# Spin‐Forbidden Excitation: A New Approach for Triggering Photopharmacological Processes with Low‐Intensity NIR Light

**DOI:** 10.1002/cptc.201700086

**Published:** 2017-06-30

**Authors:** Elham Kianfar, Dogukan Hazar Apaydin, Günther Knör

**Affiliations:** ^1^ Institute of Inorganic Chemistry Johannes Kepler University Linz (JKU) Altenbergerstrasse 69 A-4040 Linz Austria; ^2^ Institute of Physical Chemistry Johannes Kepler University Linz (JKU) Altenbergerstrasse 69 A-4040 Linz Austria

**Keywords:** controlled release, gasotransmitters, near-infrared radiation, photopharmacology, singlet–triplet absorption

## Abstract

Exposure to low‐intensity radiation in the near‐infrared (NIR) spectral region matching the optically transparent “phototherapeutic window” of biological tissues can be applied to directly populate spin‐restricted excited states of light‐responsive compounds. This unconventional and unprecedented approach is introduced herein as a new strategy to overcome some of the major unresolved problems observed in the rapidly emerging fields of photopharmacology and molecular photomedicine, where practical applications in living cells and organisms are still limited by undesired side reactions and insufficient light penetration. Water‐soluble and biocompatible metal complexes with a significant degree of spin–orbit coupling were identified as target candidates for testing our new hypothesis. As a first example, a dark‐stable manganese carbonyl complex acting as a visible‐light‐triggered CO‐releasing molecule (Photo‐CORM) is shown to be photoactivated by NIR radiation, although apparently no spectroscopically evident absorption bands are detectable in this low‐energy region. This quite remarkable effect is ascribed to a strongly restricted, but obviously not completely forbidden optical population of the lowest triplet excited state manifold of the diamagnetic complex from the singlet ground state.

Light absorption is a versatile external stimulus for triggering chemical transformations. It is therefore clear that incorporation of photoreactive compounds into cells and living organisms can offer a straightforward method for highly selective optical control over complex biochemical and physiological processes at the molecular level. This fascinating approach has the potential to spark unconventional developments in biotechnology, pharmacy and personalized precision medicine. Currently, the search for novel light‐responsive compounds and improved photoreceptor systems is a highly desirable goal of many research efforts in the context of synthetic biology, enzyme technology, photopharmacology and optogenetics.^1^

A crucial design principle for the spatiotemporal remote control over cellular functions based on biocompatible materials is an efficient long‐wavelength sensitization of their photochemical key components. More precisely, their absorption properties should extend to a significant degree into the red, far‐red or near‐infrared (NIR) regions of the electromagnetic spectrum.^2^ If this important prerequisite is not sufficiently fulfilled, many complications and side‐effects can seriously hamper practical applications of photoresponsive compounds in biological systems. It is evident that UV light has to be avoided since it is cytotoxic and will cause DNA mutations. Shorter‐wavelength radiation in the visible spectral range (<550 nm) is also not an ideal choice for most biomedical applications, since it is strongly absorbed by several cellular components and may lead to an undesired generation of toxic radicals and reactive oxygen species mediated by endogeneous photosensitizer molecules such as riboflavin.^3^

The most serious limitation for the applicability of photocontrolled reagents in living organisms is given by the low penetration depth of light into biological tissue. Viable systems should therefore be activated by photons matching the optically transparent “phototherapeutic window” of tissues with wavelengths ranging from 650 to 950 nm.^4^ By using powerful laser light sources, the challenging task of NIR‐triggered drug release has already been addressed with sophisticated nanoparticle‐based systems designed for two‐photon absorption and up‐conversion, or simply by inducing local heating steps.^5^

Herein, we introduce an unprecedented new strategy to achieve selective photocontrol at the molecular level using low‐intensity light sources for irradiation in the far‐red and NIR spectral regions. This new concept is based on the direct optical population of low‐lying excited states of light‐sensitive compounds involving absorption processes, which are formally prohibited by quantum mechanical spin selection rules (“spin‐forbidden excitation”).

As a suitable light‐dependent model reaction with significant photopharmacological relevance, we have chosen to study the controlled delivery of small amounts of carbon monoxide (CO) in response to electromagnetic radiation as an external stimulus. Systematic pharmacological strategies to investigate the physiological functions of the gasotransmitter CO by using organometallic compounds as carbon‐monoxidereleasing molecules (CORMs) were introduced 15 years ago by Roberto Motterlini and co‐workers. Many other groups followed this approach, and the promising potential of carbon monoxide to act as a therapeutic agent nowadays is well‐established.^6^

In the context of possible beneficial CO effects in photomedicine, several Group VII transition metal carbonyl complexes, including biocompatible Mn and Re based systems responsive to low‐power visible light, have been suggested as candidates for the development of photoactivated carbon‐monoxide‐releasing molecules (Photo‐CORMs).^7^ For the present study, we selected the novel dark‐stable and water‐soluble manganese complex **1** efficiently acting as a long‐wavelength photoresponsive CO‐releasing molecule (Figure 1).

**Figure 1:**
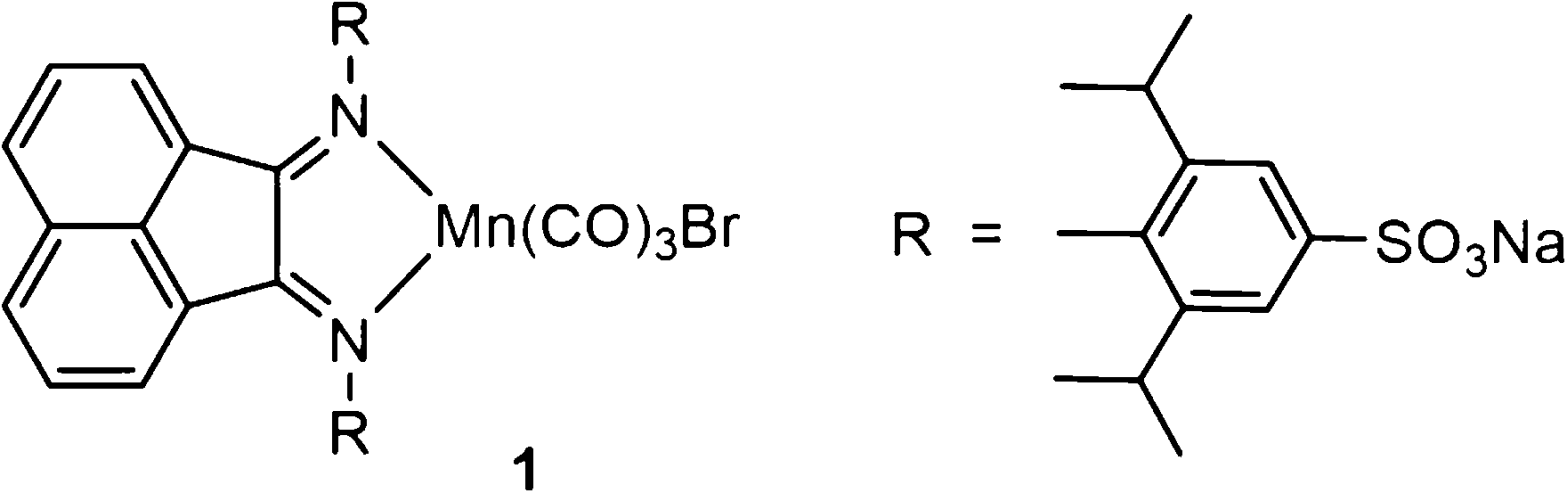
Structure of the manganese carbonyl BIAN complex **1**.

This intensely purple coloured organometallic compound of the general type *fac*‐[Mn^I^(1,2‐diimine)(CO)_3_X] (X=Br) carries a sulfonated 1,2‐diimine π‐acceptor ligand system of the bis(aryl‐imino)acenaphthene (BIAN) family.^8^ The complex displays a prominent dπ* singlet metal‐to‐ligand charge transfer (^1^MLCT) absorption band with a maximum at *λ*=513 nm dominating the visible spectral region in aqueous solution (Figure 2).

**Figure 2:**
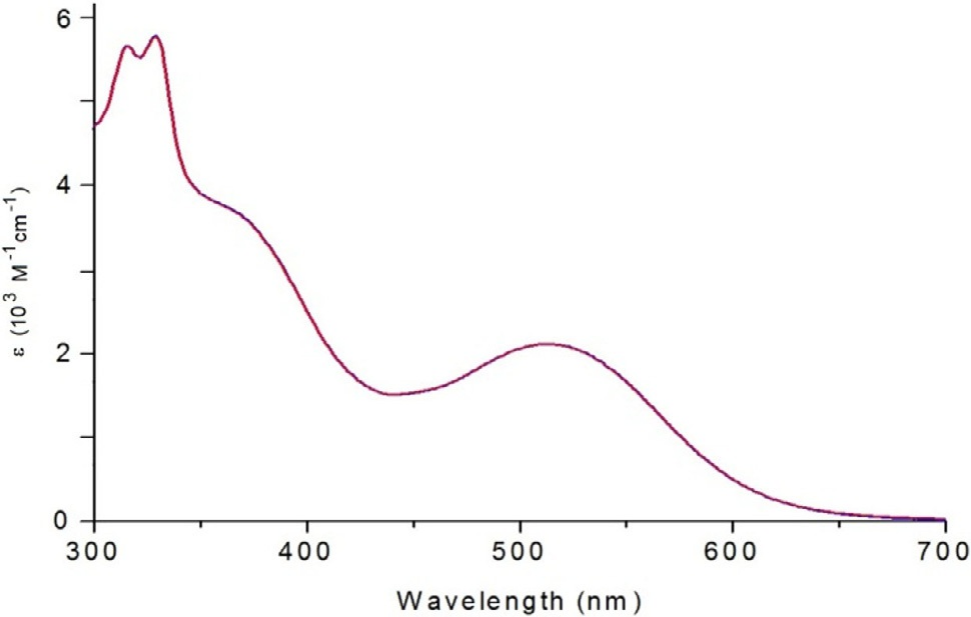
UV/Vis absorption spectra of **1** in H_2_O (1.4×10^−4^ m, 1 cm cell) recorded at *t*=0 and after *t*=80 min of storage in the dark (note that the two overlaying spectra are plotted). The lack of spectral variation clearly demonstrates the excellent thermal stability of the carbonyl complex in aqueous solution at 298 K.

The electronic spectrum of **1** is further characterized by a strong sensitivity to a decrease in solvent polarity, and the ^1^MLCT maximum is significantly red‐shifted to *λ*=568 nm in methanol. Quantitative analysis of this negative solvatochromism by a linear correlation between the solvent parameter E*_MLCT_ and the energy of the ^1^MLCT transition results in a slope of 700 cm^−1^, which confirms the charge‐transfer character of the lowest‐lying singlet excited state.^9^

Although dark samples of **1** can be stored for several hours in aqueous solution without any signs of decomposition (see Figure 2 and control experiments described in the Supporting Information), the manganese carbonyl complex undergoes a rapid photolysis upon exposure to visible light (Figure 3). Quite remarkably, the light sensitivity of **1** in the spectral region of the ^1^MLCT excited state extends to the low‐energy tail of the corresponding absorption band, matching the beginning of the first “phototherapeutic window” of mammalian tissue at around *λ*=650 nm.^4^

**Figure 3:**
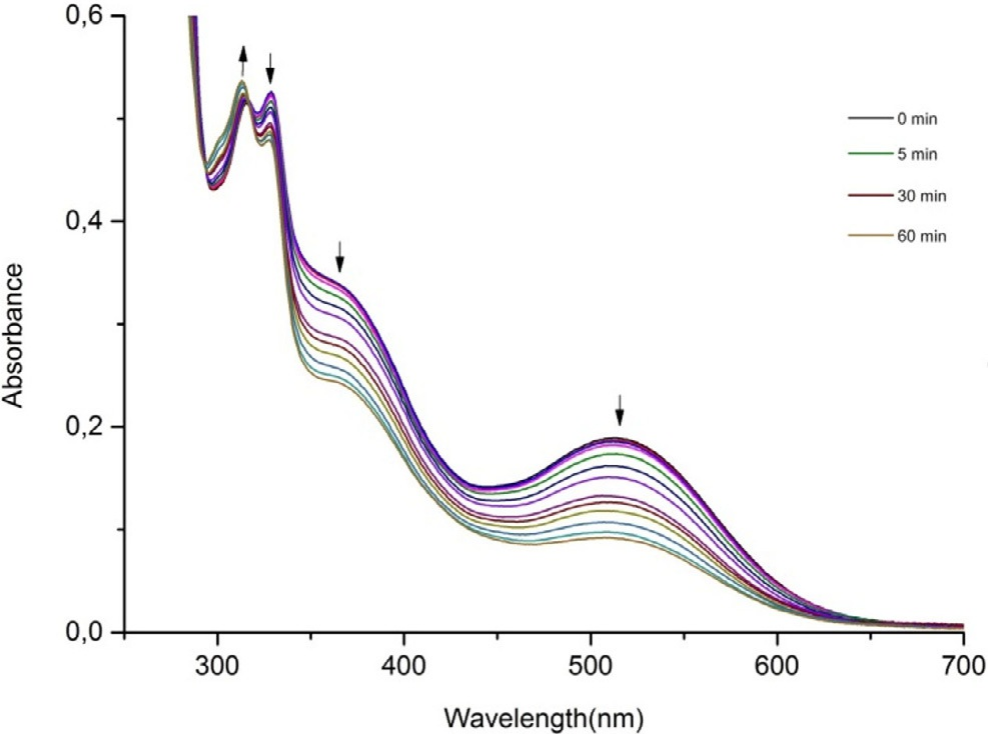
UV/Vis absorption spectral variations observed upon red‐light (>650 nm) photolysis of **1** in aqueous solution at 298 K. Spectra are shown for *t*=0, 0.5, 1, 2, 5, 10, 15, 25, 30, 35, 45, 55 and 60 min of irradiation time (Xe light source with 100 mW cm^−2^ at 650 nm, equipped with a Schott RG‐645 long‐pass filter and an IR water filter to prevent the sample from heating up).

The corresponding quantum yields of this ^1^MLCT‐induced photochemical primary reaction in aerobic aqueous solution at 298 K have been determined with monochromatic green and red light as *Φ*(525 nm)=0.54±0.07 and *Φ*(623 nm)=0.30±0.01, respectively. It is important to note that the quantum yield value obtained in the red‐light tail of the charge‐transfer absorption band is sensitive to the presence of oxygen and increases by more than 25 % to a value of *Φ*(Ar, 623 nm)=0.38±0.03 in argon‐saturated solution (see the Supporting Information). Such an initially unexpected behaviour indicates a potential direct contribution of the energetically low‐lying triplet‐excited‐state manifold to the observed photoreactivity, since excited states with a triplet spin multiplicity are expected to be significantly quenched in the presence of triplet dioxygen (^3^O_2_) from air and thus faster deactivated in competition with photoproduct formation.

The spectral variations shown in Figure 3 are accompanied by a change of the characteristic strong signals in the carbonyl stretching vibration region as monitored by FTIR spectroscopy (Figure 4).

**Figure 4:**
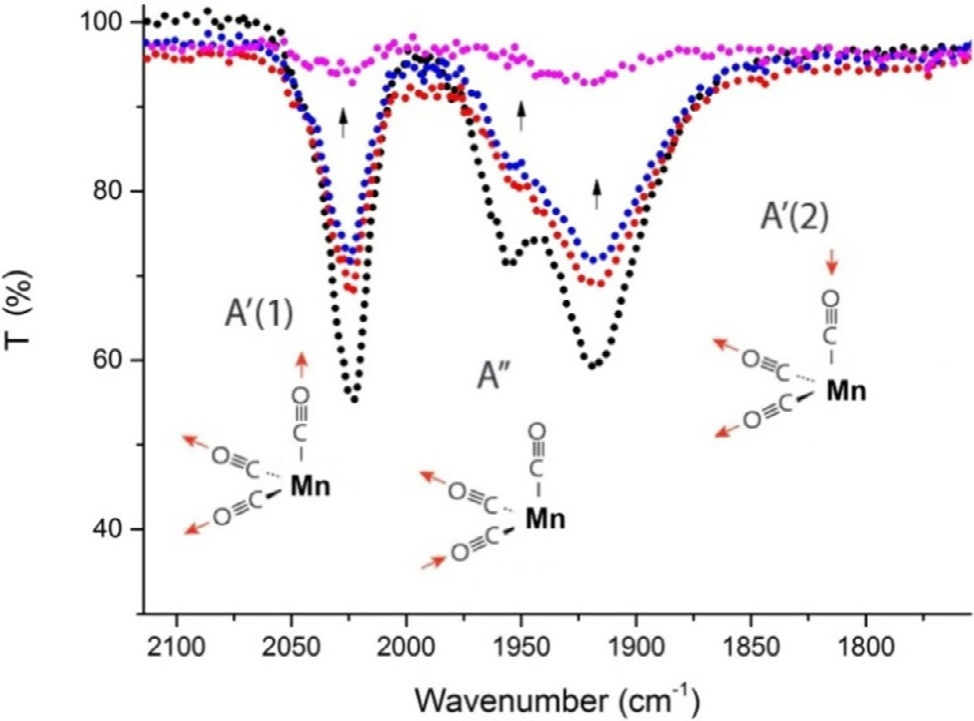
Gradual loss of ν(CO) infrared stretching vibration signals of complex **1** in the course of photoproduct formation observed upon exposure to ambient light. Data for *t*=0, 20, 50 min, and for long‐term irradiation of the compound are shown.

Pseudooctahedral [Mn^I^(1,2‐diimine)(CO)_3_X] derivatives with a facial (*fac*) arrangement of their carbonyl ligands typically display three strong CO stretching vibrations in the IR spectral range of 1900–2040 cm^−1^. The highest frequency ν(CO) band, observed at 2023 cm^−1^ for complex **1** (Figure 4), corresponds to the totally symmetric in‐phase vibration assigned as A′(1).^10^ In addition, two lower‐energetic IR bands denoted as A′′ and A′(2) appear at 1954 cm^−1^ and 1918 cm^−1^, respectively. All of these IR bands vanish upon prolonged visible‐light exposure of the tricarbonyl complex **1**, which strongly indicates complete CO loss from the manganese centre. Interestingly, in the initial phase of the photolysis, the asymmetric A′′ vibration of the equatorial CO ligands at 1954 cm^−1^ disappears, while the remaining two ν(CO) signals involving the axial CO ligand are still observable. This might indicate that one of the equatorial CO ligands is lost in the primary photochemical step. It should also be noted that after light exposure, the primary photoproduct of **1** is no longer thermally stable in aqueous solution, and the remaining CO signals gradually disappear even in the dark.

To further characterize the light‐triggered CO‐releasing steps, the headspace gas composition of sealed samples of complex **1** in aqueous solution was analysed in the course of the primary period of the photolysis (Supporting Information). In these experiments, which follow the first few minutes of the decomposition reactions shown in Figure 3 and Figure 4, an increase of the gas‐phase concentration of carbon monoxide was detected and quantitatively analysed by FTIR measurements with a direct spectroscopic method as previously described in more detail.7b, 11

It turned out that indeed only one CO molecule per Mn centre is lost in the photochemical primary step. Green (*λ*=525 nm) or red (623 nm) LED light sources were applied for triggering the CO‐release mechanism under controlled conditions. With a typical sample (Figure 5) containing 0.3 mg of **1** in aqueous solution, at 298 K about 100 nmol of free carbon monoxide were obtained within less than 5 min of LED irradiation in the spectral region of the ^1^MLCT band (30 % conversion assuming a 1:1 ratio of CO to manganese). Further details about quantum yield determinations and all irradiation experiments are given in the Supporting Information.

**Figure 5:**
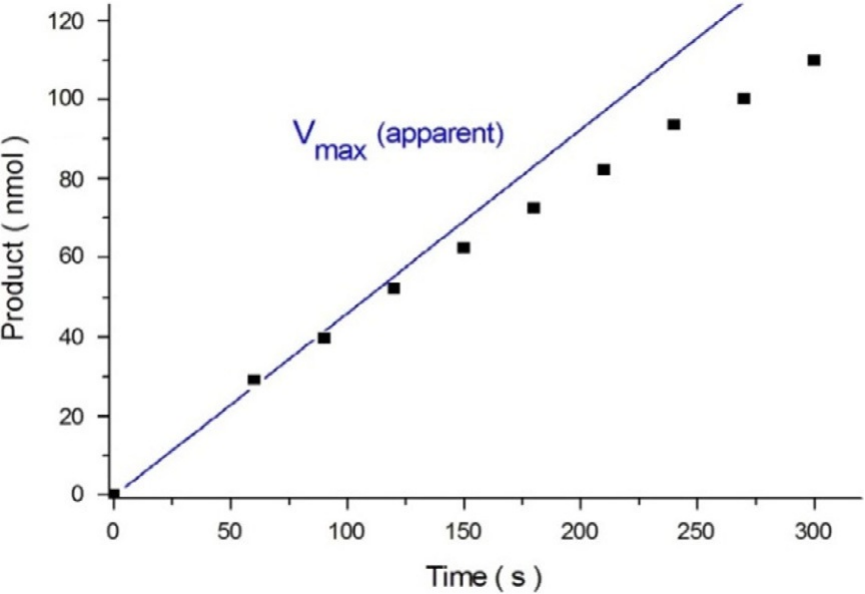
Determination of the initial rate (apparent *V*_max_ value) of photoproduct formation for steady‐state irradiation of a solution of **1** (0.3 mg) in H_2_O (1.5×10^−4^ m, 525 nm LED, 298 K).

In this context it should be pointed out that the natural activity of heme oxygenase enzymes responsible for the controlled endogeneous release of the gasotransmitter carbon monoxide inside living cells and tissues in a biological environment^6^ is typically in the activity range of around 1 nmol CO h^−1^ mg^−1^,^12^ which is 5000 times lower than the CO release rate obtained with Photo‐CORM **1** under the visible‐light irradiation conditions reported in Figure 5 (apparent *V*_max_=0.43±0.01 nmol s^−1^). For the development of light‐controlled enzyme model compounds (artificial photoenzymes) acting as a photopharmacological substitute of the natural heme oxygenase/CO system,1b it is therefore necessary to reduce the light sensitivity described above for the manganese complex **1** by about three to four orders of magnitude to reach the physiologically relevant levels of carbon monoxide release. Moreover, it would also be very advantageous to use ambient daylight as an external stimulus to maintain an appropriate intracellular level of CO concentration for beneficial effects,^6^ and to expand the sensitivity of the carbon‐monoxide‐releasing molecule significantly further into the far‐red and NIR spectral regions of the spectrum, which is an important feature for enabling potential applications in deep‐tissue photomedicine such as reaching specific subcutaneous target regions from outside.^4^ We could show that for compound **1** these basic requirements can be readily provided by exploiting the unprecedented approach of “spin‐forbidden excitation” in the low‐energy spectral region, as will be described in the following section.

Irradiation experiments of the manganese carbonyl complex **1** in aqueous solution were also carried out in a dark room on an optical bench with polychromatic far‐red and near‐infrared light using a Xe lamp equipped with a water‐filled infrared filter and a NIR‐transparent long‐pass colour glass filter (Schott RG‐715). Under these conditions, only light within the so‐called “first phototherapeutic optical tissue window” with wavelengths from *λ*=720 to 950 nm could reach the samples. Although dark control references did not show any changes, an exposure to low‐energy NIR radiation induced the same kind of spectral variations of the Photo‐CORM **1** as observed before upon visible‐light photolysis (Figure 3; see also the Supporting Information). Since the long‐wavelength ^1^MLCT absorption band of **1** corresponding to the first excited singlet state (S_1_) of the compound does not extend into the NIR spectral region (Figure 2), a different excitation mechanism generating the photochemically active species has to be postulated when all photons with λ<700 nm are excluded. We have clearly shown that even a 780 nm long‐pass filter still gives rise to the same type of photoreaction (Supporting Information), which cannot be ascribed to inhomogeneous broadening of the spin‐allowed charge‐transfer absorption band. Moreover, compared to a regular ^1^MLCT irradiation with visible light, this additional excitation pathway was found to require significantly longer irradiation times for the same level of conversion at a given light intensity and sample concentration. Such a behavior is not surprising since most but obviously not all of the NIR light is passing through the sample without being absorbed. As schematically depicted in Figure 6, the direct population of the lowest triplet excited state manifold (T_1_) of complex **1** from the corresponding ground‐state energy level (S_0_) was identified as a plausible explanation for this unusual phenomenon.

**Figure 6:**
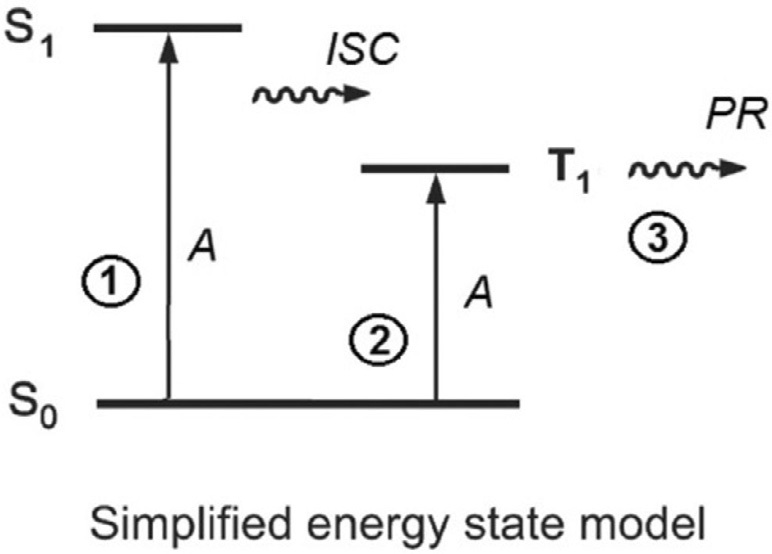
Jablonski diagram illustrating the concept of spin‐forbidden excitation (A: absorption, ISC: intersystem crossing, PR: photoreaction). The lowest‐energy triplet state levels (T_1_) responsible for product formation (pathway 3) can either be reached from the first excited singlet state (S_1_) followed by intersystem crossing (pathway 1), or may be directly generated by singlet–triplet absorption (pathway 2).

Since such an optical singlet–triplet transition (Figure 6, pathway 2) is a spin‐prohibited process, it will only occur with a very low probability. Therefore, in molecular systems with a negligible degree of spin–orbit coupling (SOC), the corresponding absorption bands are expected to be extremely weak with small extinction coefficient values (typically *ϵ*<1 m ^−1^ cm^−1^) and thus are not easy to identify in the electronic spectra. Nevertheless, in the solid state, where the concentration of the absorbing species reaches a maximum value, such spin‐restricted electronic transitions have been unambiguously characterized in several cases.^13^ In solution, clear evidence for direct singlet–triplet absorption of phosphorescent compounds can be obtained by analysing the luminescence excitation spectra.^14^ In nonluminescent systems, however, the detection of weak but clearly resolved T_1_←S_0_ absorption bands by UV/Vis/NIR spectroscopy requires a significant degree of spin–orbit coupling. Such a perturbation of the quantum mechanical selection rule of spin conservation can be induced by the so‐called “heavy atom effect” (an impact of elements with higher atomic numbers *Z* resulting in a larger SOC parameter *ξ*), which has been demonstrated to further enhance the virtually “forbidden” singlet–triplet absorption bands in intensity.^15^

In the case of carbonyl complex **1**, the presence of the elements manganese (*Z=*25) and bromine (*Z=*35) should be able to induce a noticeable degree of spin–orbit coupling,^16^ which could then enhance the probability of a direct singlet–triplet absorption process. Despite all efforts using cuvettes with longer optical path‐lengths and higher substance concentrations, in aqueous solution we were not able to unambiguously identify any well‐resolved NIR bands indicating such singlet–triplet absorption bands. In acetonitrile solution, however, we could obtain additional weak signals in the spectral region of the “phototherapeutic window”, which corroborates the hypothesis that spin‐forbidden excitation is responsible for the NIR‐light‐induced photoreactivity of complex **1** (see the Supporting Information). The corresponding singlet–triplet splitting Δ*E*(S_1_–T_1_)=2800 cm^−1^ is in agreement with the small energy‐gap range typically observed for luminescent metal complexes with a significant MLCT‐excited‐state character.^17^ In addition, for related 1,2‐diimine carbonyl complexes carrying the heavier third‐row transition metals tungsten (*Z=*74) or rhenium (*Z=*75), similar low‐energy absorption bands have recently been observed and assigned to ^3^MLCT (T_1_←S_0_) transitions in their UV/Vis/NIR and phosphorescence excitation spectra.^18^

No attempts were made to measure a reliable quantum yield for the NIR photolysis of complex **1** in aqueous solution. From the energy state model shown in Figure 6, however, a reasonable estimate of *Φ*(NIR) can be derived. Since the quantum yield of an intersystem crossing (ISC) process will never exceed 100 %, it can be concluded that in the absence of other deactivation processes, the photoproduct formation quantum yield following direct spin‐forbidden excitation (pathway 2) should be equal or larger than the experimentally accessible quantum‐yield values obtained for the spin‐allowed excitation (pathway 1). In other words, the reduced apparent rate of photoproduct generation observed upon NIR‐light exposure of **1** (Supporting Information) is due to the much smaller absorption cross‐section of the samples in the region of the singlet–triplet absorption bands, but not due to a lower intrinsic quantum yield of the photochemical primary process (pathway 3).

For the sake of completeness, it should also be pointed out here that the simplified Jablonski energy‐level diagram given in Figure 6 illustrating the basic strategy of spin‐forbidden excitation does not fully account for the complex nature of the lowest excited electronic triplet state manifold (“T_1_ state”). This is especially true in the case of metal complexes including **1**, where important aspects such as the degree of admixture of higher‐lying electronic states, charge‐transfer perturbations in the wavefunctions of the triplet states and a pronounced zero‐field splitting into three sublevels have to be included for a detailed analysis.^10^, ^19^ Such a more in‐depth photophysical discussion, however, was out of the scope of the present work.

In summary, we have shown here for the first time that by exploiting the possibility of direct “spin‐forbidden excitation” of coordination compounds, a nonsensitized photochemical reaction can be triggered with radiation in the far‐red and NIR spectral regions. This previously neglected activation strategy is well suited for low‐power light control over biologically and photopharmacologically relevant processes. Therefore it has a promising potential for innovative applications in deep‐tissue photomedicine, as demonstrated here with a novel Photo‐CORM system for the controlled release of the gasotransmitter carbon monoxide. Further examples based on this new approach are currently under investigation.

## Conflict of interest

*The authors declare no conflict of interest*.

## Supporting information

As a service to our authors and readers, this journal provides supporting information supplied by the authors. Such materials are peer reviewed and may be re‐organized for online delivery, but are not copy‐edited or typeset. Technical support issues arising from supporting information (other than missing files) should be addressed to the authors.

Supplementary
