# Supplementary material for: Spin‐Forbidden Excitation: A New Approach for Triggering Photopharmacological Processes with Low‐Intensity NIR Light
Source: ChemPhotoChem. 2017 Jun 30;1(9):378–82. doi: 10.1002/cptc.201700086 (PMC5658980; doi:10.1002/cptc.201700086)
Supplement: Supplementary file 1 — Supplementary [file CPTC-1-378-s001.pdf]

CHEMISTRY OF LIGHT INTERACTION
CHEMPHOTOCHEM
ACROSS THE WHOLE SPECTRUM

Supporting Information

Spin-Forbidden Excitation: A New Approach for Triggering Photopharmacological Processes with Low-Intensity NIR Light

Elham Kianfar,[a] Dogukan Hazar Apaydin,[b] and Günther Knör*[a]

cptc_201700086_sm_miscellaneous_information.pdf

Table of Contents

Materials and Methods S2
Results and Discussion S2-S12
Synthesis of complex **1** S2
Solvatochromism S4
Electronic transitions S5
Irradiation conditions S5-S7
Photochemical experiments S7-S9
Carbon monoxide detection S10-S11
Quantum yield measurements S11-S12
Thermal stability S13-S14
References S15
Author Contributions S15

Materials and Methods

All starting chemicals, if not otherwise stated, were commercially available and used as supplied. Water was purified with a Milli-Q system (Millipore, Bedford, MA, USA). Other solvents used were of spectrograde quality (ethanol, acetonitrile) or purified according to standard procedures. NMR spectra were recorded in D_2O on a Bruker Digital Avance III (300 MHz) spectrometer. ^{1}H-NMR shifts are reported in ppm relative to $Si(CH_3)_4$. For UV-Vis-NIR absorption measurements up to 1100 nm, a Varian Cary 50 spectrophotometer was used. FTIR spectra from the solid materials were recorded in the range of 4000 - 400 cm^{-1} on a Shimadzu IR-Affinity-1 spectrometer equipped with a Specac Golden Gate single reflection diamond ATR accessory. Detection of photoinduced CO release into the headspace gas phase was performed with a BRUKER IFS 66/S FTIR spectrometer or on a Thermo Scientific Nicolet IS5 spectrometer in 10 cm gas cells. For quantitative analysis of the carbon monoxide concentration over time, measurements were compared to a standard calibration gas mixture containing 1 vol% of CO [1]. Mass spectra were obtained on an autoflex III smartbeam broker (MALDI-TOF) mass spectrometer. The samples were measured with or without matrix (dithranol as matrix 100:10 of sample) in positive and negative mode.

Results and Discussion

Synthesis of complex **1**:

The sulfonated water soluble bis(arylimino)acenaphthene ligand 2,6-iPr$_2$-4-sodiumsulfonato-C$_6$H$_2$-BIAN was prepared from acenaphthenquinone and the sodium salt of 2,6-diisopropyl sulfanilic acid in anology to literature methods [2].
Preparation of the water soluble manganese complex *fac*-Mn^I(2,6-iPr$_2$-4-sodiumsulfonato-C$_6$H$_2$-BIAN)(CO)$_3$Br (**1**) was carried out under exclusion of light as follows:
55 mg (0.078 mmol) of the sulfonated BIAN-ligand were dissolved in dry methanol and added to a solution of $Mn(CO)_5Br$ (21.45 mg) in dry methanol. The mixture was stirred for 28 hours at room temperature under nitrogen atmosphere. The solvent was then removed and the deeply purple coloured solid residue was dried in vacuo and kept under nitrogen atmosphere and exclusion of light.

1: $C_{39}H_{38}N_2O_9S_2Na_2MnBr$, M = 923.68 g/mol

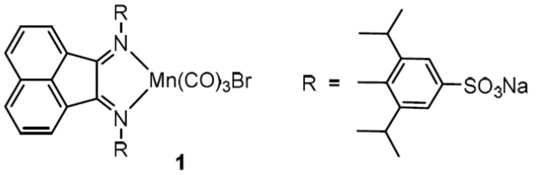

1

^{1}H-NMR (300 MHz, D_2O, 25 °C, TMS, δ[ppm]): 8.16 (d, 2H), 7.93 (s, 2H), 7.81 (s, 2H), 7.50 (t, 2H), 6.70 (d, 2H), 3.06 (m, 2H), 2.85 (m, 2H), 1.39 (d, 6H), 1.26 (d, 6H), 1.05 (d, 6H), 0.50 (d, 6H)

IR: 1918 cm^{-1}, 1954 cm^{-1}, 2023 cm^{-1}; ν(SO): 1182 cm^{-1}

MS: with matrix, negative mode: $[M-2Na^+ +H_2O]^{2-}$ = 448.
with matrix, positive mode: $[M+Li]^{2+}$ = 465.5

UV-Vis (H_2O, 298K): λ_{max} = 513 nm (ε = 2112 $M^{-1}cm^{-1}$), 329 nm, 316 nm.

WILEY-VCH

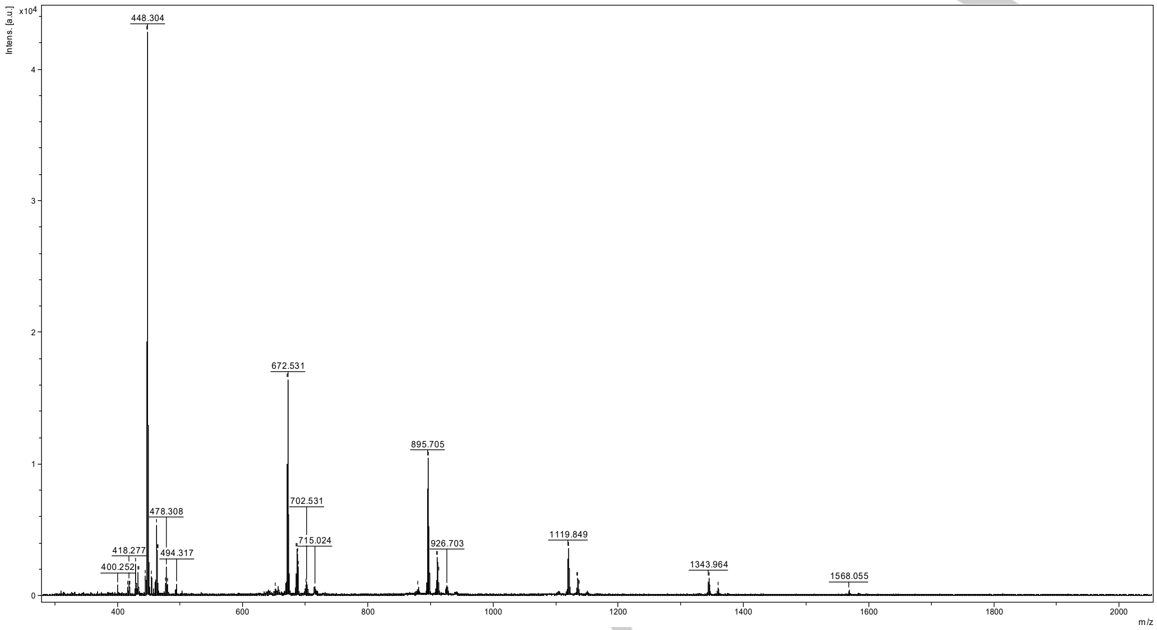

MALDI-TOF mass spectrum of complex **1** in negative ion mode showing a $[M\text{-}2Na^{+} + H_2O]^{2-}$ = 448 m/z main peak and matrix adducts.

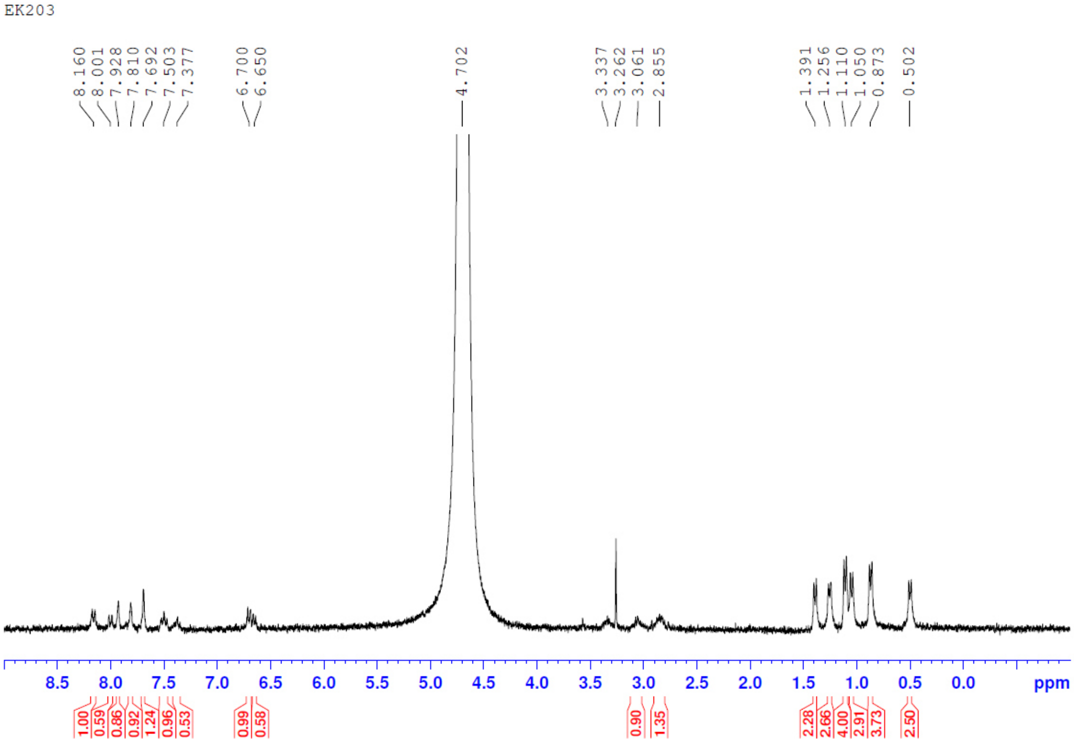

^{1}H-NMR spectrum of partially photolyzed complex **1** in D_2O (brown glass NMR tubes do not prevent actinic light exposure)

Solvatochromism:

By decreasing the solvent polarity from water to other solvents, the charge transfer band maximum of the Mn carbonyl complex **1** is shifted to longer wavelengths (Fig. S1).

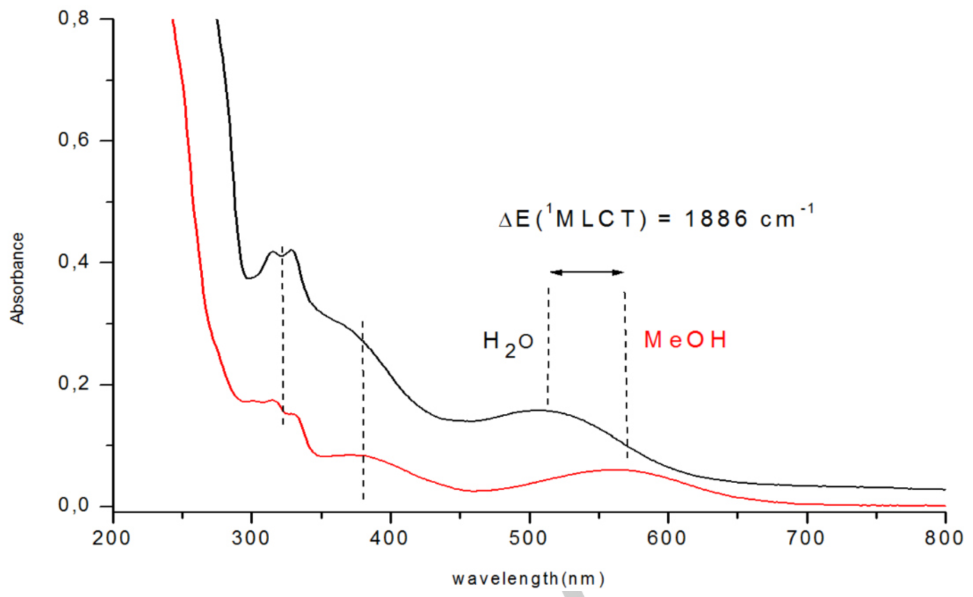

Figure S1: Bathochromic-shift of the visible absorption maximum (MLCT band) of **1** upon decreasing solvent polarity (negative solvatochromism). Note that the other absorption maxima are not influenced in energy by this solvent change.

The degree of negative solvatochromism can be quantified by plotting the energy of the MLCT maximum against the solvent polarity parameter E^*_{MLCT} [3] as shown in Fig. S2 below.

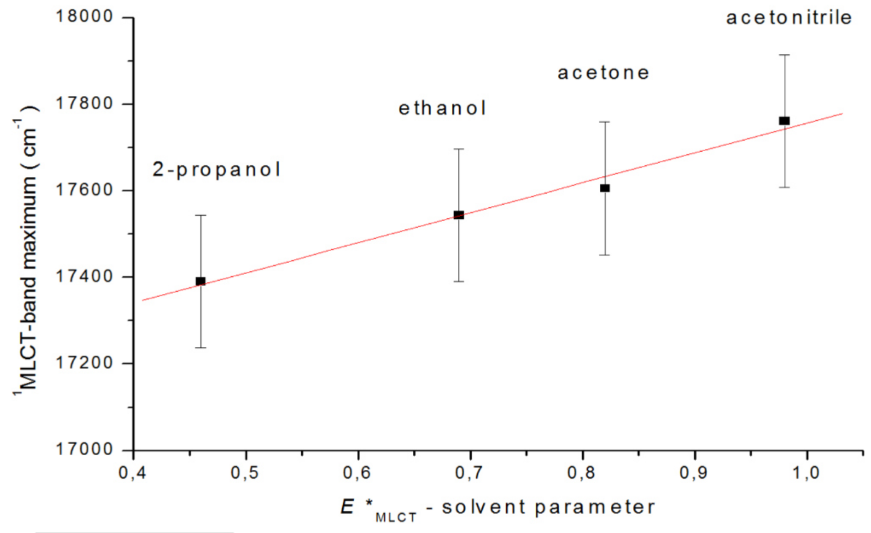

Figure S2: Bathochromic-shift of the charge transfer band of **1** in different solvents.

Electronic transitions:

In the ultraviolet spectral region, intense intraligand bands involving the π-electron system of the BIAN-moiety dominate the absorption spectrum of the manganese carbonyl complex **1** (Fig S3 a). The chromophoric ^{1}MLCT absorption band of **1** in the visible spectral region corresponding to the spin-allowed population of the first excited singlet state (S_1) of the compound does not significantly extend into the far-red and NIR-spectral region. In non-aqueous solution, however, additional weak electronic transitions of **1** could be identified at lower energies outside the visible spectrum (Fig. S3). Quite similar spectral features have been reported before for structurally related rhenium carbonyl complexes, although such a highly resolved fine structure could not be observed in solution due to the occurrence of rather weak and inhomogeneously broadened signals [4].

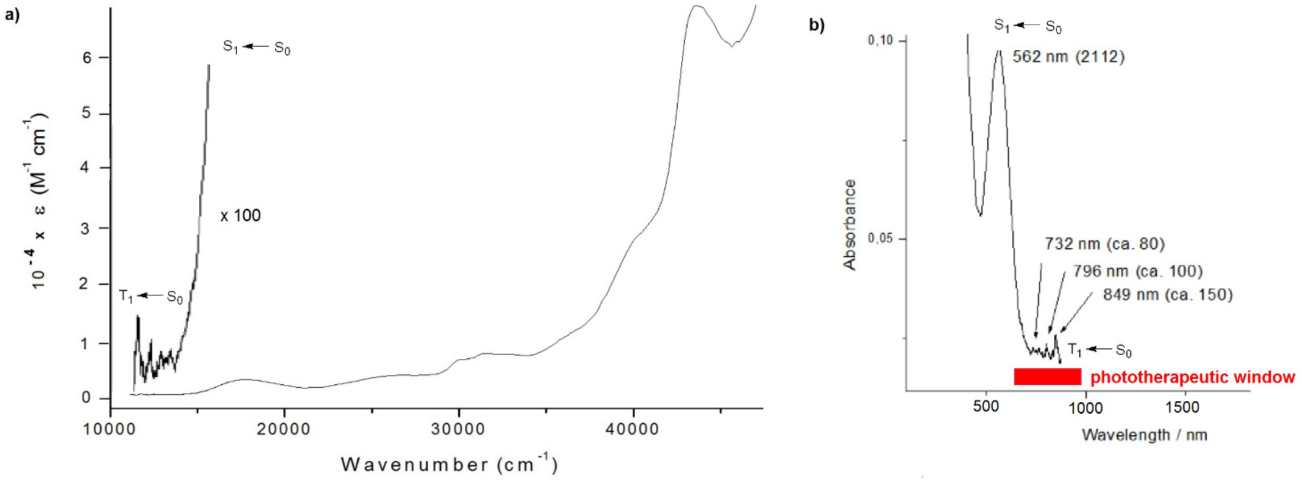

Figure S3: UV-VIS-NIR absorption spectra of complex **1** in acetonitrile solution plotted with increasing energy (left side) and increasing wavelength (right side). The spectral region of the spin-forbidden transitions (scaled up by a factor of 100) matches the optically transparent far-red and NIR-region of mammalian tissue (the so-called first "phototherapeutic window") as symbolized with the red bar on the right side of the plot covering 650 nm - 950 nm.

Irradiation conditions:

Excitation in a specific visible spectral region such as green- and red-light exposure of samples (Fig. S4) was carried out with LED light sources. ALUSTAR 3W LED-Modules (LEDxON) commercially available (Conrad Electronics) mounted with precise lens optics into an Al-case with 10° output angle were used. The ready-to-use system is based on the EDISON Opto Edixeon S single color high-power LED series (http://www.edison.opto.com.tw).

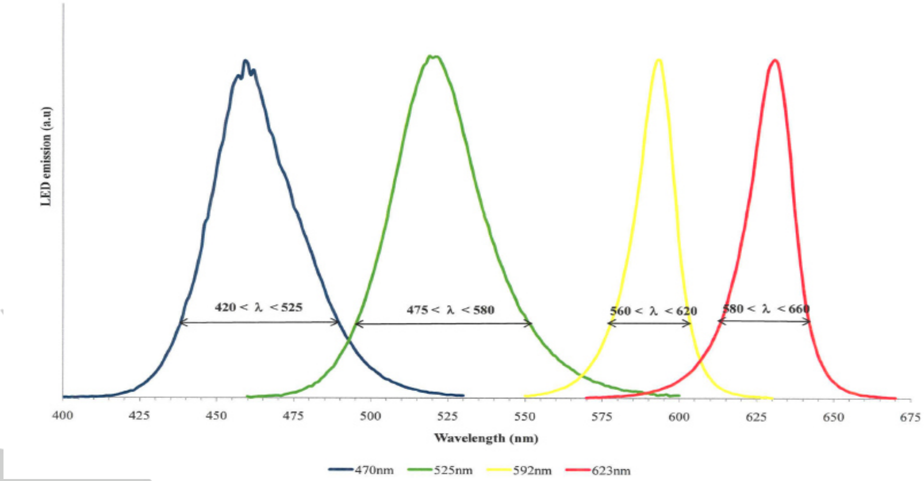

Figure S4: Emission spectra of the LEDs used for irradiation and quantum yield determination (maxima: green LED 525 nm, red LED at 623 nm)

Visible light photolysis experiments were also carried out on an optical bench setup with a 150 W Xenon lamp (Osram Model XBO 150W/1), an Oriel Newport universal arc lamp housing equipped with an Aspherab UV-grade fused silica condenser lens and an AMKO IR liquid filter filled with water (80 mm light pass). Different color glass filters were applied to cut off the shorter wavelength range of the lamp output (Fig. S5, S6). The power supply was set to 100 W (5.6 A; 17.7 V) for all experiments. A calibrated spectroradiometer (Luzchem SPR-4001) was used to characterize the spectra The full spectral characteristics of the excitation source is displayed below.

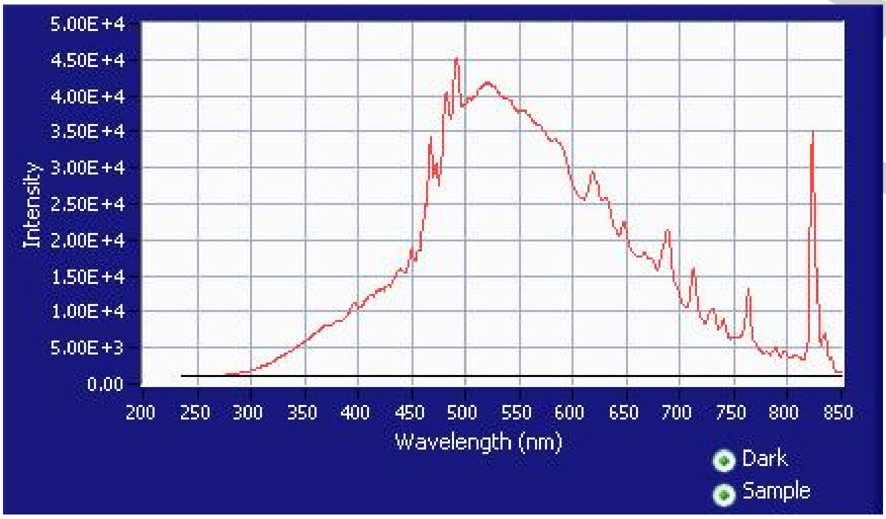

Figure S5: Unfiltered spectral output of the OSRAM XBO 150W/1 light source (Xenon lamp) monitored with the Luzchem SPR-4001 spectroradiometer.

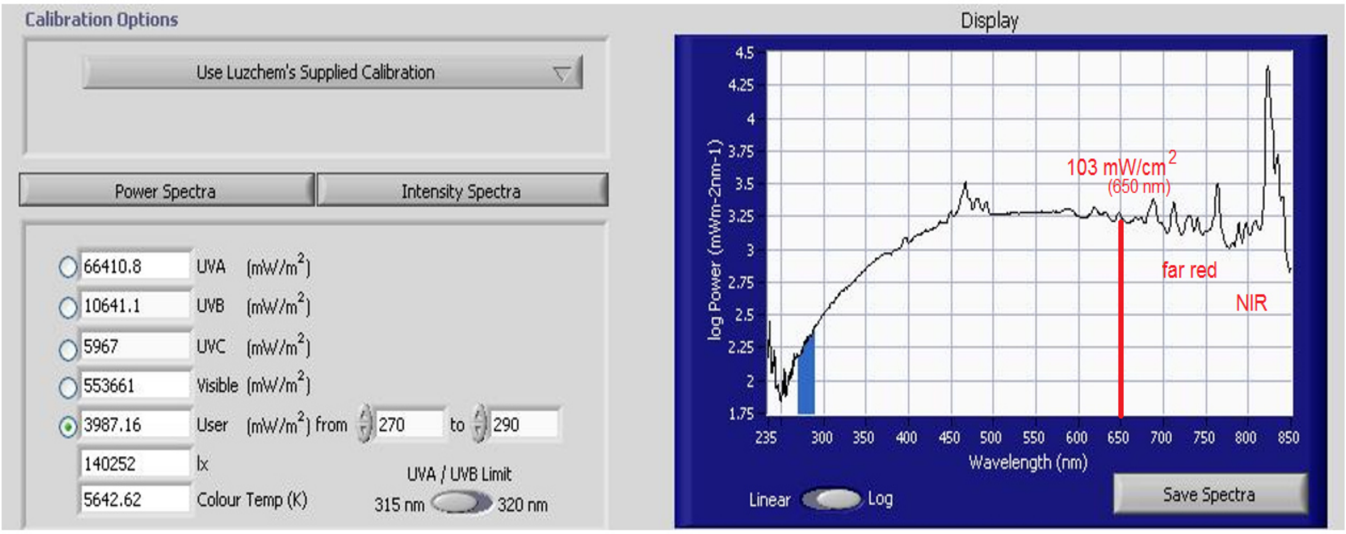

Figure S6: Wavelength dependent Xe-lamp flux density monitored with the Luzchem SPR-4001 spectroradiometer (ca. 100 mW/cm^2 at 650 nm).

To test out the longer wavelength photoreactivity of complex **1** in the far-red and NIR-spectral regions, samples were also irradiated on the optical bench setup with a 150 W xenon lamp as described above for the visible light photolysis experiments. Additional far-red and/or NIR-transparent (50 mm x 50 mm square) long-pass filters (e.g. RG 715, RG 780) were placed in front of the sample cuvettes as for the individual experiments. In Figure S7, the transmission range of the filter glass RG 715 is shown as an example.

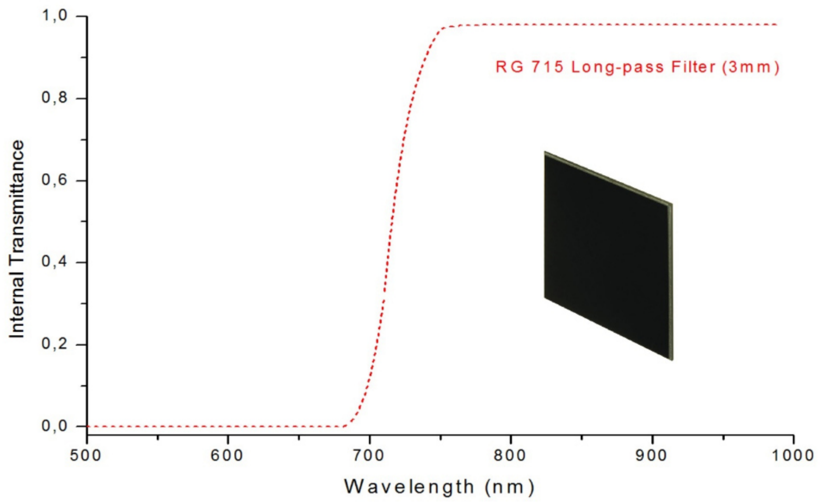

Figure S7: Transmittance curve of the far-red light transparent colour glass filter Schott RG715. A similar short-wavelength excitation light filtering effect, however with an inflection point at 780 nm, is characterizing the internal transmittance curve of Schott RG780 long-pass filter used for NIR-irradiation.

Photochemical experiments:

Spectral variations observed when the manganese carbonyl complex **1** was irradiated with green (525 nm), yellow (592 nm) and red (623 nm) LED light are shown in the following figures.

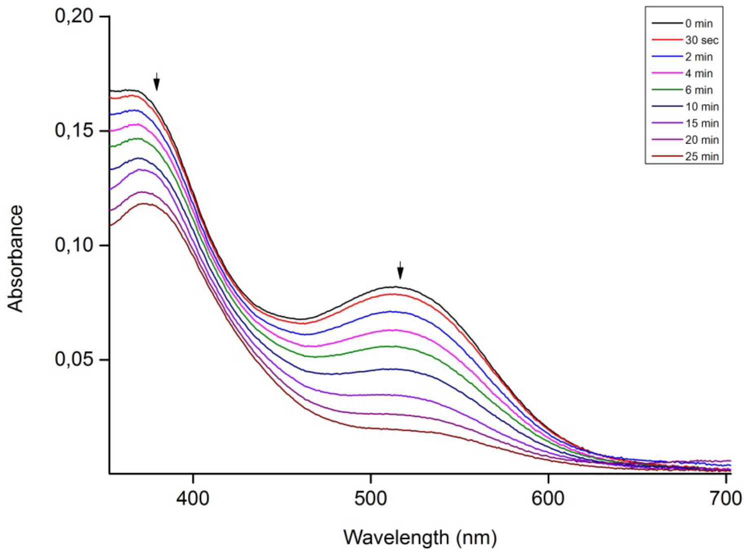

Figure S8: Photolysis of compound **1** in aqueous solution with green LED light (298 K, 1 cm cell, t= 0-25 min at 525 nm).

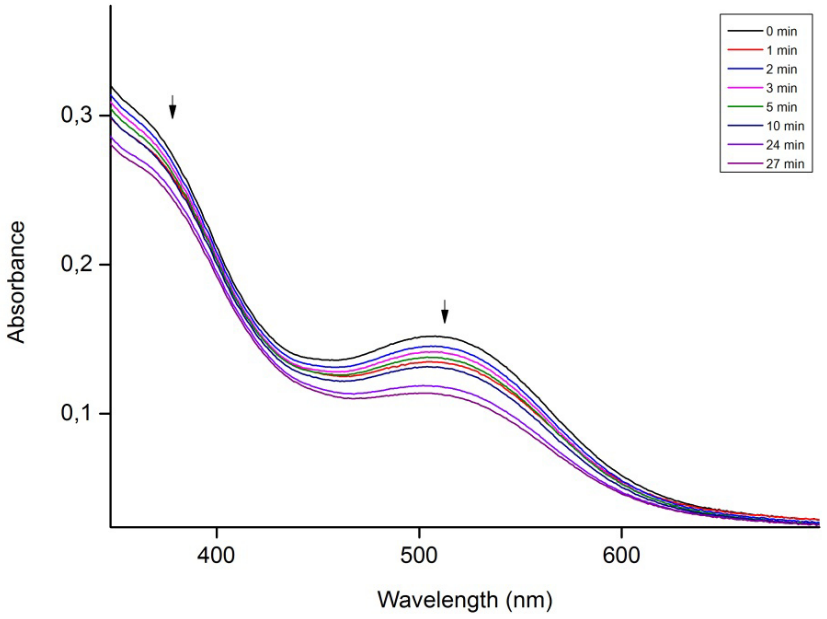

Figure S9: Photolysis of compound **1** in aqueous solution with yellow LED light (298 K, 1 cm cell, t= 0-27 min at 592 nm).

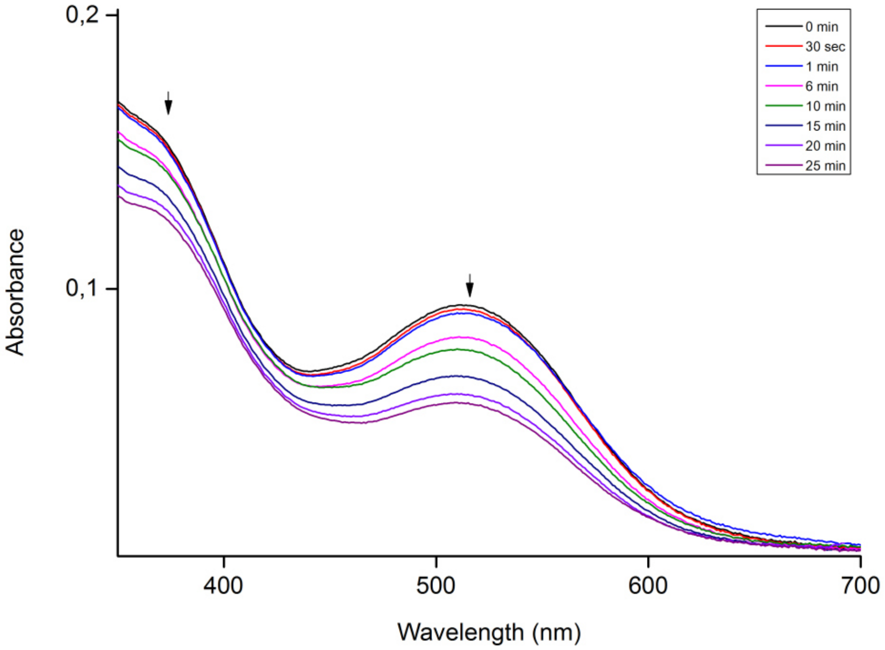

Figure S10: Photolysis of compound **1** in aqueous solution with red LED light (298 K, 1 cm cell, t= 0-25 min at 623 nm).

The corresponding spectral variations induced by polychromatic far-red and NIR light excitation of **1** in aqueous solution excluding all wavelengths shorter than 715 nm or 780 nm are given in Figures S11 and S12:

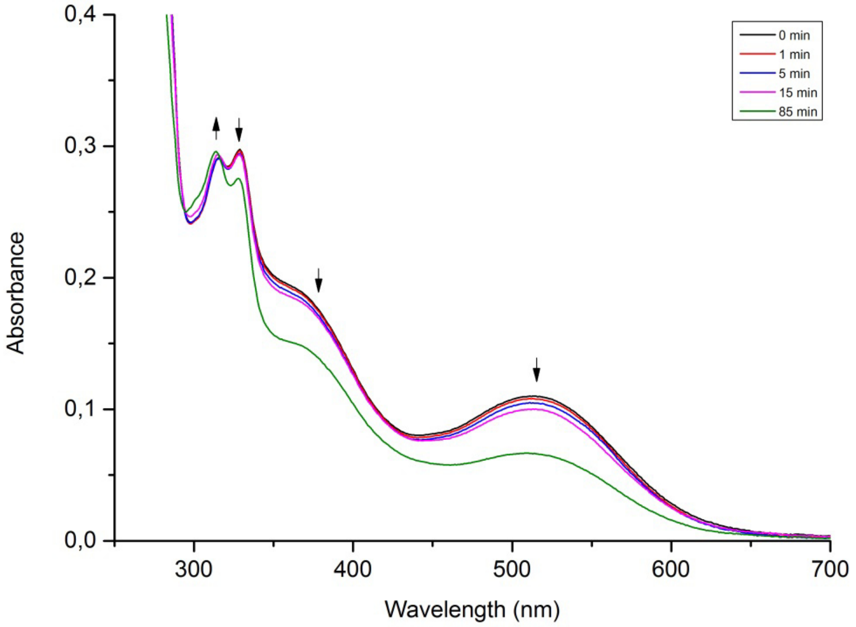

Figure S11: Photolysis of compound **1** in aqueous solution with Xe-lamp light (298 K, 1 cm cell, t= 0-85 min, RG715 long-pass filter).

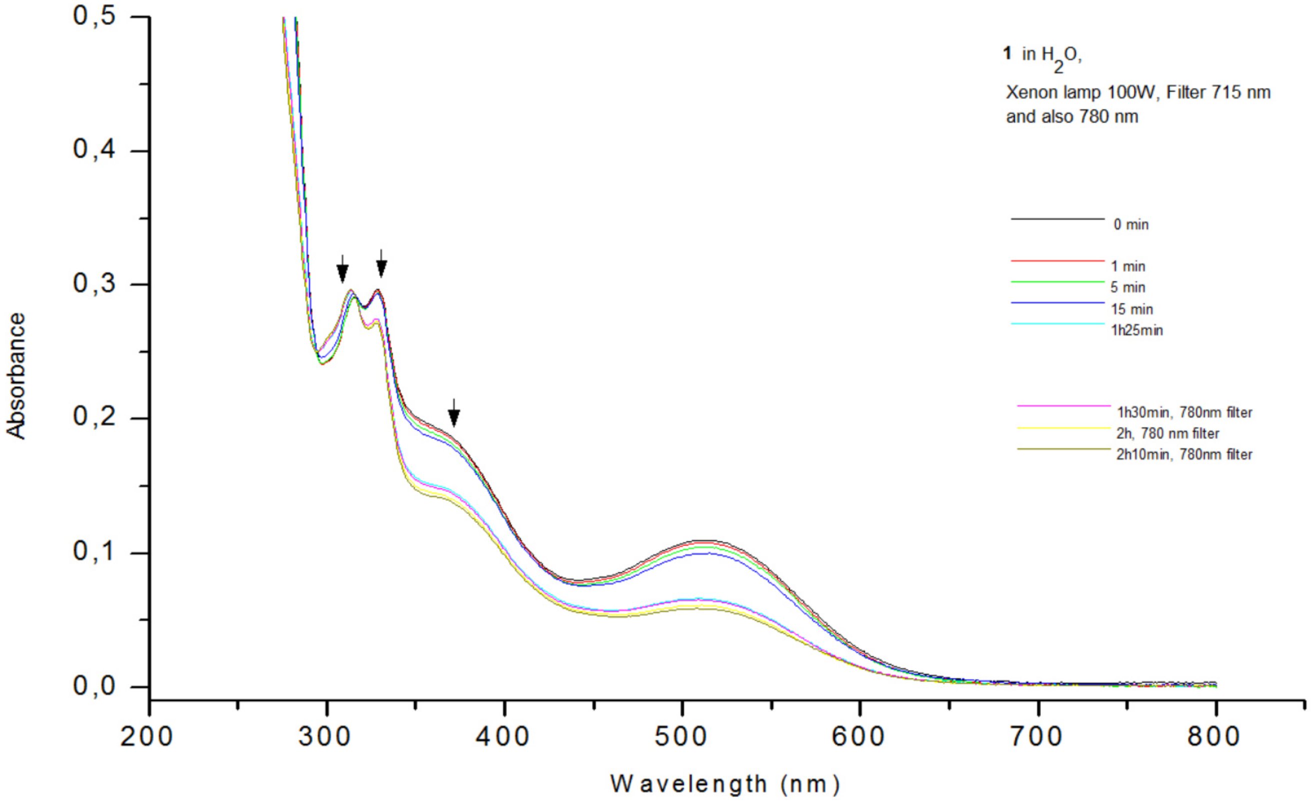

Figure S12: NIR-photolysis of compound **1** in aqueous solution with Xe-lamp light (298 K, 1 cm cell, RG715 and then also RG780 long-pass filters used).

Carbon monoxide detection:

The release of CO was monitored by measuring the vibration-rotation line spectrum of carbon monoxide in the gas phase with the characteristic P and R branches centered around a gap at 2143 cm^{-1} (Fig. S13). Gas samples were taken with a syringe from the headspace of photolyzed solutions and transferred into a gas tight transmission cell designed to measure IR absorption in transmission mode (ZnSe windows, 10 cm pathlength). For quantification of the reactions, a defined amount of calibration gas was used [5].

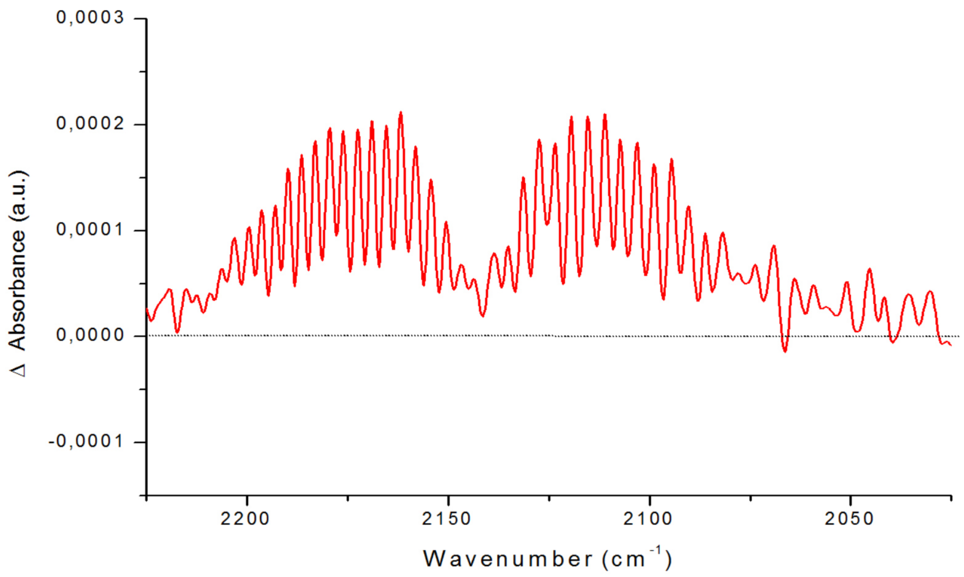

Figure S13: FTIR-difference spectrum of a diluted photoreaction gas-mixture indicating the increase of carbon monoxide concentration compared to the corresponding dark control sample.

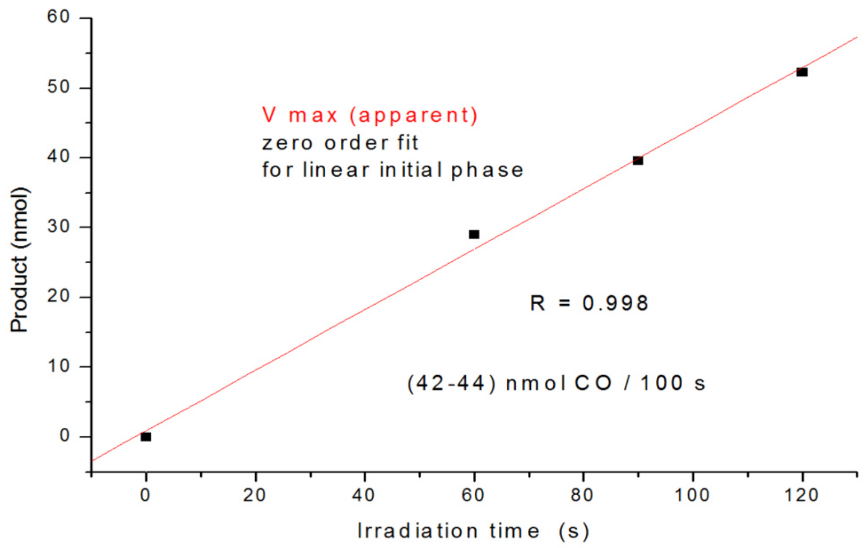

Figure S14: Determination of the carbon monoxide release rate induced by steady-state visible light irradiation of a solution of 0.3 mg **1** in H_2O (1.5 × 10^{-4} M, 525 nm LED, 298 K), assuming that one CO ligand of complex **1** is lost in the photochemical primary process (see main text of the paper for discussion).

For quantifying the total amount of CO released after a certain irradiation time, the sample solutions were prepared as follows:

2.0 ml of aqueous stock solution of manganese carbonyl complex **1** (c = 1.49 × 10^{-4} M) were filled into a septum-capped cuvette with 3.1 ml of headspace volume above the solution. After photolysis of the solution with the selected LED for a defined irradiation time, a headspace gas sample (1.0 ml) was drawn and introduced into the FTIR measurement cell. A dark-control solution was also prepared with identical conditions and kept unexposed to light before the CO detection measurement (same temperature and time interval as with the irradiated sample). The total amount of the photochemically released carbon monoxide from the complex **1** is taken as the sum of CO accumulated in the headspace gas and the small CO amount remaining in solution. The number of CO molecules dissolved in water at 298 K was estimated by using Henry's law [6]. This latter amount did not contribute significantly to the total amount of CO detected as shown below.

In a typical experiment, using a 525 nm LED for irradiation in the region of the visible-light absorption maximum (^{1}MLCT-band) of the manganese carbonyl complex **1**, after 5 min of photolysis in aqueous solution at 298 K, the increase of CO concentration measured corresponds to a total amount of about 110 nmol of photoreleased carbon dioxide (Table S1).

Table S1. Controlled visible-light induced release of carbon monoxide with manganese carbonyl complex **1** in water at 298K

CO in the headspace [a]	CO in solution[b]	Total amount of CO released	Irradiation wavelength	Irradiation time
1.1 × 10^{-7} mol	7.1 × 10^{-14} mol	110 nmol	525 nm	300 s

[a] Quantified by FTIR difference spectroscopy; [b] Calculated by Henry´s law

Quantum yield measurements:

A home-built optical bench mounted system producing an electrical signal proportional to the photon flux absorbed by the investigated samples was used for exact conversion quantum yield determinations.The technical details of this setup were reported and tested recently [7]. The system allows for a convenient absolute radiative power detection with a calibrated solar cell detector. The setup is equipped with a turning wheel for rapid switching between four different permanently installed high-power LED light sources covering the visible spectral range for photolysis experiments. The spectral output of the built-in LEDs (470, 525, 592 and 623 nm), which are of the same type as those used for visible-light LED illumination experiments described before is shown in Fig. S4.

Calibration of the home-built system was carried out by applying classical chemical actinometry with Reinecke´s salt in dilute acidic solution according to the recommended standard procedures [8,9] (0.01 M: φ = 0.31 at 470nm; 0.04M: φ = 0.29 at 525nm; 0.01M: φ = 0.27 at 592nm; 0.025M: φ = 0.29 at 623nm).

Results obtained fror green and red light irradiation of the manganese carbonyl complex **1** are given below in Table S2:

Table S2. Photoreaction quantum yields for irradiated solutions of the manganese complex **1** in water at 298K [a]

Irradiation conditions	φ (1 min)	φ (2 min)	φ (3 min)	φ (4 min)	φ (5 min)	φ (average)
525 nm LED, air	0.46	0.52	0.53	0.56	0.61	0.54 ± 0.07
525 nm LED, Ar	0.48	0.47	0.47	0.47	0.47	0.47 ± 0.01
623 nm LED, air	0.29	0.29	0.31	0.31	0.30	0.30 ± 0.01
623 nm LED, Ar	0.41	0.39	0.37	0.36	0.35	0.38 ± 0.03

[a] Individual values calculated for an increasing degree of conversion in 1 min steps in the linear regime of photolysis

Experiments were carried out at 298 K in septum-capped gas-tight quartz cuvettes (1 cm pathlength). Samples were continuously stirred during the 1 min photolysis intervals.The spectral changes for the quantum yield determination measurements reported in Table S2 are given below (Fig. S15 and Fig. S16):

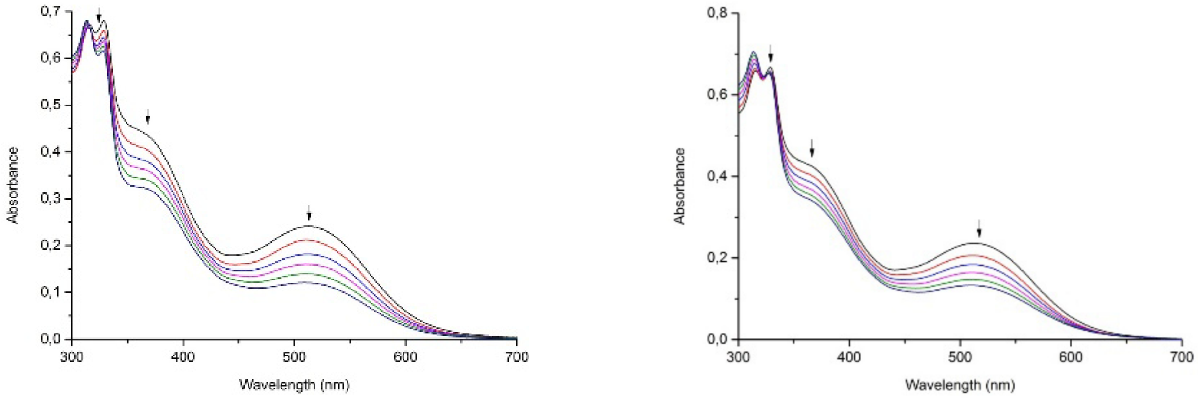

Figure S15: Photolysis of complex **1** in the quantum counter setup using the 525 nm LED as a light source (c = 1.13 × 10^{-4} M in H_2O, V = 2.0 ml, t = 0, 1, 2, 3, 4, and 5 min irradiation times shown). The curves on the left side are recorded under aerobic conditions, the curves on the right side with a solutuion purged with Ar for 10 min.

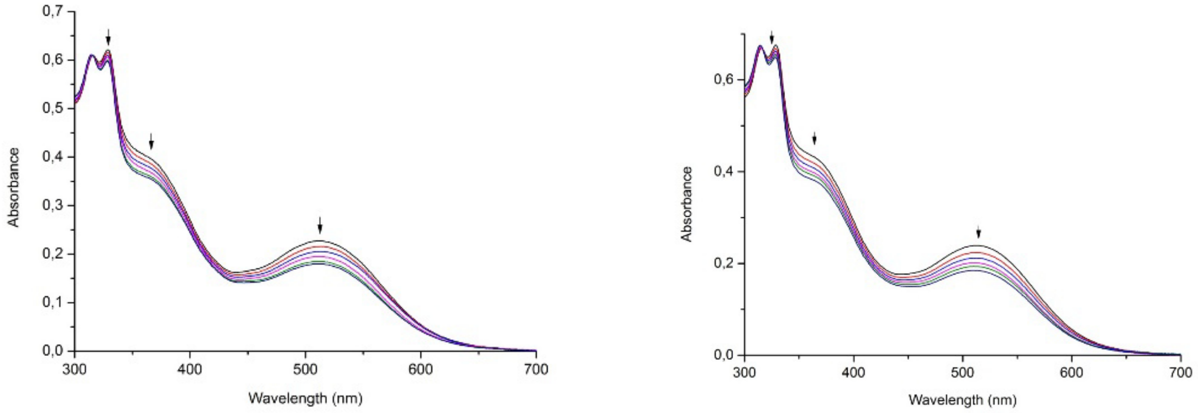

Figure S16: Photolysis of complex **1** in the quantum counter setup using the 623 nm LED as a light source (c = 1.13 × 10^{-4} M in H_2O, V = 2.0 ml, t = 0, 1, 2, 3, 4, and 5 min irradiation times shown). The curves on the left side are recorded under aerobic conditions, the curves on the right side with a solutuion purged with Ar for 10 min.

Thermal stability:

The control experiments for dark-samples of complex **1** in aqueous solution at 298 K (as tested in the CO-releasing measurements described in the previous sections) did not show any production of free carbon monoxide. Together with the lack of UV-Vis spectral variations, this clearly indicates, that despite of a conspicuous light sensitivity of the carbonyl compound, the dark stability of **1** in aqueous solution is excellent.

Even after 5 hours kept under exclusion of light (gas-tight sample cuvettes wrapped with Al-foil), there was no CO detected from the headspace of aqueous solutions of the manganese carbonyl complex **1** (1.5×10^{-4} M in water at 298 K). When the primary photolysis was initiated, however, the photoproduct formed (assumed to have lost one equatorial CO ligand as discussed in the main text of the paper) is no longer long-term stable in the dark. These secondary processes have not been fully elucidated yet.

Some of the relevant observations on the thermal stability of **1** after the generation of the primary photoproducts are briefly reported below.

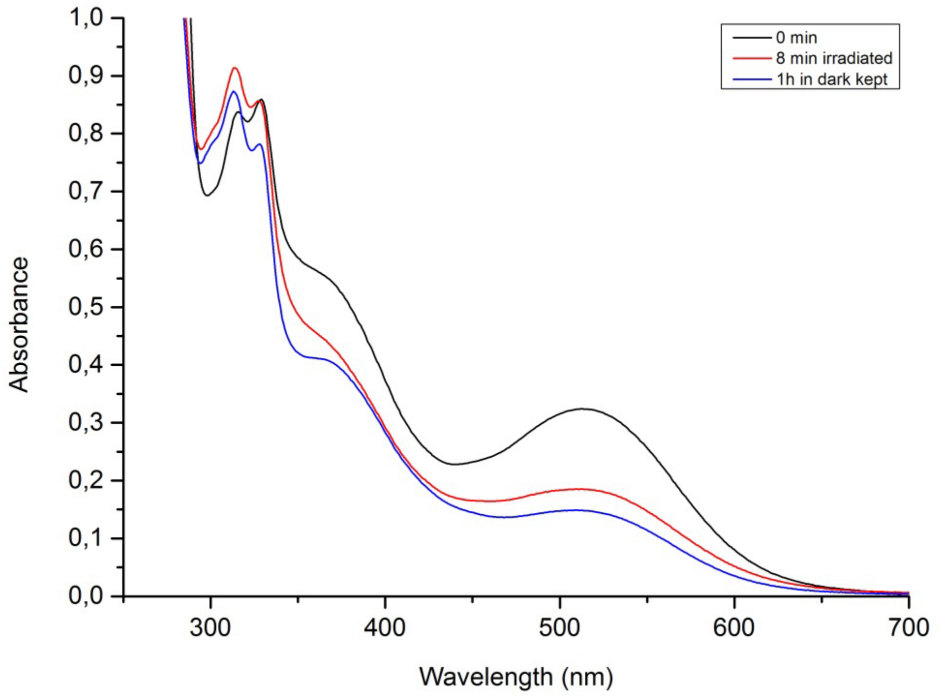

Figure S17: Photolysis and light-initiated secondary reactions of complex **1** using the 525 nm LED excitation source (c = 1.48×10^{-4} M in Ar-purged H_2O at 298 K, Spectra for t = 0 and t = 8 min irradiation times are shown together with the final absorption spectrum after 1 h of dark reaction.

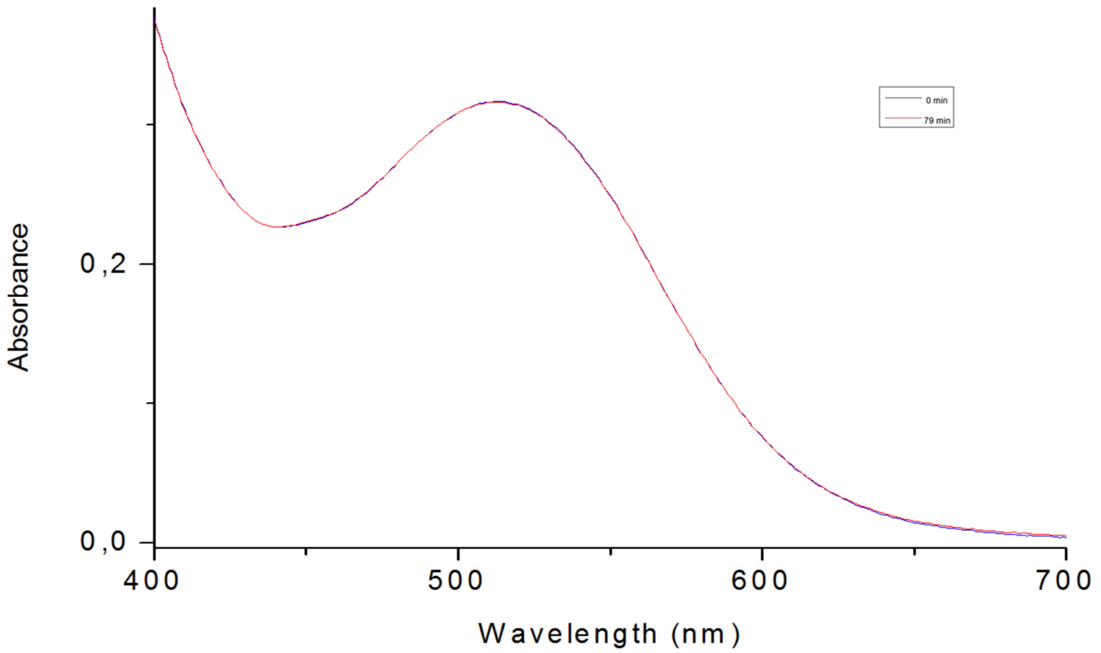

Figure S18: Visible spectra of a solution corresponding to the experiment shown in Fig. S17, but exclusively kept in the dark for about the same total time (79 min) before measurement of the second curve. In contrast to the light exposed sample, no dark reaction is observable and the absorption spectrum remains completely unaffected (see Fig. S17 for comparison).

WILEY

References

[1] E. Portenkirchner, E. Kianfar, N. S. Sariciftci, G. Knör, *ChemSusChem* **2014**, *7*, 1347-1351.

[2] E. Kianfar, U. Monkowius, E. Portenkirchner, G. Knör, *Z. Naturforsch.* **2014**, *69b*, 691-698.

[3] D. M. Manuta, A. J. Lees, *Inorg. Chem.* **1983**, *22*, 3825-3828.

[4] F. W. M. Vanhelmont, M. V. Rajasekharan, H. U. Güdel, S. C. Capelli, J. Hauser, H.-B. Bürgi, *J. Chem. Soc., Dalton Trans.* **1998**, 2893-2900.

[5] E. Kianfar, C. Schäfer, M. R. Lornejad-Schäfer, E. Portenkirchner, G. Knör, *Inorg. Chim. Acta* **2015**, *435*, 174-177, and references cited therein.

[6] R. Sander, *Atmos. Chem. Phys.* **2015**, *15*, 4399-4981.

[7] K. T. Oppelt, E. Wöß, M. Stiftinger, W. Schöfberger, W. Buchberger, G. Knör, *Inorg. Chem.* **2013**, *52*, 11910-11922.

[8] H. J. Kuhn, S. E. Braslavsky, R. Schmidt, Chemical Actinometry, IUPAC Technical Report 2004 (http://www.iupac.org/publications/pac/76/12/2105/pdf/);

[9] M. Montalti, A. Credi, L. Prodi, M. T. Gandolfi, *Handbook of Photochemistry*, 3rd Ed., CRC Taylor & Francis, Boca Raton, **2006**, p. 609, and references cited therein.

Author Contributions

E. K. prepared the compounds and conducted the measurements. D. H. A. contributed to this work by detecting the gas phase CO concentrations. G. K. acquired funding, administered the project, suggested the concept, planned the experiments, interpreted the results and wrote the paper. All authors contributed to analyzing the data, discussing and improving the manuscript.

WILEY-VCH
